# Opportunities and challenges of mainstreaming disaster risk management in faith institutions

**DOI:** 10.4102/jamba.v16i1.1667

**Published:** 2024-05-17

**Authors:** Peter Masvotore

**Affiliations:** 1 Research Institute for Theology and Religion, College of Human Sciences, University of South Africa, Pretoria, South Africa

**Keywords:** challenges, disaster risk management, mainstreaming, opportunities, united theological college, Zimbabwe

## Abstract

The coronavirus disease 2019 (COVID-19) epidemic had created mayhem world over in the year 2020 and confirmed the need and urgency of equipping religious leaders with disaster risk management (DRM) knowledge and skills. Religious leaders are often expected to be among the first responders when disasters strike. This is especially the case in Africa where faith communities often have greater reach among the populace than the state itself. Using multifaceted methodology and purposive sampling interview analysis, this article shows that the responses of religious leaders in Africa, Zimbabwe in particular, to COVID-19 highlighted the urgency of adopting a more focussed and deliberate approach towards equipping religious leaders with DRM knowledge and skills. It further demonstrates that in some areas religious leaders responded effectively (communicating the right message, impact mitigation activities, etc.), in most other areas, they were as unprepared and in a state of paralysis as the rest of the populace. It would however, be grossly unfair to critique their response since most of them have never been exposed to the basic tenets of DRM, either in their formation or as part of life-long learning.

**Contribution:**

The article concludes by suggesting mainstreaming tragedy hazard reducing in the curricula of religious institutions for stoppage, mitigation and actual answer to current and future tragedies within communities.

## Introduction

Africans and Africa from pre-colonial history through colonialism, slavery, economic exploitation, wars of independence, civil wars, famine, poverty, unemployment, pandemics such as human immunodeficiency virus (HIV) and acquired immunodeficiency syndrome (AIDS), floods, in addition to new coronavirus disease 2019 (COVID-19), have been facing disasters in various ways. Africans have learned to adapt to all these challenges and consequently any new crisis that comes is likely to be treated as others. Even when her people are threatened by death, Africa remains a hopeful continent. Katenda (2022:3) declared that, with Africa emersed in sins of recklessness, syncretism, bad governance and disorganised public administration in certain places, Africa and Africans still remain hopeful because they are full of determination and hope.

The question this article tries to answer is how do theological institutions prepare and equip religious leaders in disaster risk management (DRM)? Further to that, how do Africans and Zimbabweans see this current crisis of COVID-19? Do they view it as other crises before it and those that will come after it? Coming closer home Zimbabwe is a non-coastal poor but no longer a food basket nation in southern Africa. This nation has faced a number of encounters from the late 1990s that have depressingly impacted its sustenance condition. Landlocked countries experience climate change such as melting of glaciers that is critical to water resources, landslides and reduction of biodiversity among other disasters like drought desertification among others. Amid these encounters are prevalent scarcity, HIV and AIDS, unemployment, money challenges, persistently changing climatic conditions, tremors, cyclones, financial and political uncertainty as well as the COVID-19 pandemic; altogether these have attributed to continuous shortages of food (WFP 2010:3).

Coronavirus disease 2019 pandemic that had caused mayhem globally in 2020, confirmed the urgency of training religious leaders with DRM knowledge and skills. Religious leaders are often anticipated to be among the first responders when disasters strike. This is predominantly the case in Africa where faith communities frequently have greater reach among the populace than the state itself. The reasons as to why this is so shall be explored in detail in the research elsewhere. Using multifaceted methodology and purposive sampling interview analysis, this article demonstrates that the responses of religious leaders in Africa and Zimbabwe to COVID-19 emphasised the urgency of espousing a more intensive and thoughtful approach towards equipping religious leaders with DRM knowledge and skills. The article concludes by suggesting mainstreaming disaster risk reduction (DRR) in the curricula of religious institutions for prevention, mitigation and effective response to current and future disasters within communities.

## Defining disaster and disaster risk management

A disaster is a fundamental challenge that happens for a quick or extended period and inflicts extensive anthropological, physical, financial or ecological damage, which surpasses the capability of the affected community or village to handle it by means of their locally available resources (WHO/EHA 2002:4). In other words, where above 95% of demises are instigated by perils occurring in rising nations, such hazards are declared disasters (Brown 1996). There is no doubt that where disasters occur, they alter the course of history as witnessed in the Chimanimani area in Zimbabwe during the Cyclone Idai that took place in March 2019. Lives were lost, the environment was damaged and the situation changed at the blink of an eye. Muhlanga, Muzingili and Mpambela (2019:47) explain a disaster as, ‘any catastrophic occurrence stanching from actions such as quakes, torrents, disastrous accidents, fires, or detonations that causes great damage or loss of life’. They further describe a disaster (Muhlanga et al. 2019):

As a serious disruption of the functioning of a community or a society at any scale due to hazardous events interacting with conditions of exposure, vulnerability and capacity leading to human, material, economic and environmental losses, and impacts. (p. 47)

This shows that where a disaster strikes, it has a negative impact on both humans and economy and even the environment.

One question that needs to be answered in the process of defining disasters is when is an event a disaster? According to Koresawa (2010:3), when more than 20% of the inhabitants are affected and need backup support or their residential units have been demolished is considered a disaster. He further indicates that where more than 10 persons are acknowledged to have been killed and a call for international assistance is requested or where more than 100 persons are revealed to have been affected it is considered a disaster. Nielsen and Lidstone (1998) aver that where at least 40% of the means of livelihood are destroyed, for example cars and sources of income assets, major roads, infrastructure, bridges, telecommunications, it can be declared a disaster by the State.

For Landesman (2001:3), disasters can be categorised into:

- A minor tragedy that initially affects limited societies, which then requires support outside the affected public.
- An extensive adversity that disturbs a humanity, which then needs nationwide or global support.
- Recurrent and sporadic catastrophes that hinge on the likelihood of existence and have the reoccurrence history of an assumed hazard and its influences.
- A slow-onset disaster such as draught, increase desertification, widespread diseases.
- An unexpected-onset catastrophe that is prompted by a dangerous incident that arises rapidly or unpredictably.
- Abrupt-onset tragedies related to earthquakes, volcanic explosions, ostentatious overflows, biochemical eruptions, life-threatening building catastrophes or transportation fatalities.
- Artificial calamities, which are the result of scientific or humanoid perils. Instances include stampedes (2000 World Cup qualifier between Zimbabwe and South Africa where 12 lives of supporters were lost at the National Sports Stadium) (Chikamhi 2020 Herald news), fire flames, road fatalities, work-related accidents, lubricant tumbles and atomic bursts and/or radiation.
- Conflict and calculated attacks.

On the other hand, when talking about disaster peril administration, it denotes the implementation of tragedy risk lessening strategies and approaches, preventing novel calamity risks, reducing prevailing tragedy risks, as well as managing remaining risks, contributory to the consolidation of pliability and decrease of fatalities (Dube & Munsaka 2018:4). Communities in Zimbabwe, particularly in low lying areas, face disaster risks of various kinds, and people should actively reduce or mitigate against these risks. Deliberate actions taken to reduce risks and reduce losses that some communities suffer from disasters are what is called DRM. In a progressively ‘risky’ community, Beck noted that:

Emergency management is firmly situated in policy and public discourse as an essential system for protection, preparedness, and response to contemporary threats. (1992:2)

In risk management, there is need to ask action questions like, even though we know the drought is coming, what can we do to make the effect of the drought less vicious on our people? Even though we know that Muzarabani (one of the low-lying areas located alongside Mozambique and Zimbabwe boarder in Mashonaland Central area) will be flooded when the Zambezi overflows, what can we do to minimise the losses that our people there suffer because of the flooding? As we have heard on National News that a cyclone is approaching, what can we do to minimise our losses because of the cyclone? Answering these questions is partaking in DRM.

## Methodology

Using a multifaceted methodology and purposive sampling method, the study focussed on the United Theological College (UTC),^1^ which is one of the biggest ecumenical colleges in Africa and selected 50 participants inclusive of administration, student union, participating Heads of denominations and other students who have graduated from the college and are in the areas where disasters are happening. The research included both male and female participants of the age of 18 years and above. Both WhatsApp and telephone interviews were conducted during the study. Both WhatsApp and telephonic interviews were conducted keeping in mind such graduate students who were in areas that were inaccessible because of destruction of roads and bridges and therefore travelling to these areas was a nightmare. These interviews became a viable option in speeding up the process. Two focus group discussions (FGDs) were also conducted, and these became a useful tool for data collection. The data was analysed to generate ideas on opportunities and challenges for mainstreaming DRM in the curriculum of theological and religious institutions with reference to the UTC.

All ethical clearance considerations were followed. A permission letter was obtained from the college to conduct interviews with students and staff members. Informed consent was obtained from the participants to interview them and assuring them that the information was for educational purposes, and no financial benefit was extended. The participants were also assured of confidentiality through the use of pseudonyms.

## Theoretical framework

To bring this study into context, the investigator used Social Capital theory as the theoretical framework:

A theoretical framework is an intangible method of showing the way a scholar makes reasonable logic of the connection between numerous aspects that were recognized as significant to the problem. (Sekaran 2000:2; see also Kolanchu 2011:24)

The reason to choose this theory is inspired by the fact that individuals who reside in the community participate in detecting and come up with ways of how to solve challenges bedevilling the community. Furthermore, it is driven by the empowerment niche of resourcing local people who deliberately come together to have an impact to the society. According to Kolanchu (2011):

Community consciousness prepares individuals for communal exertions in responding to crises. This buttresses a proverb that goes ‘Knowledge is Power’ that consequently, bring community responsiveness as a significant aspect of tragedy risk decrease by equipping the community that is facing risks. (p. 24)

Nielsen and Lidstone (1998:18) also argued that academia is the expansion of data and skills to inform the populace and enable them to make informed conclusions. Claridge (2004:8) avers that this theory, also looks at the connectedness between diverse people, the value of social networks and the attachment of similar people, with norms of mutual understanding, is considered a suitable theoretic lens for this kind of research, to form an academic context. Additionally, this theory undoubtedly relates very well with community consciousness because their aim is to improve the wellbeing of the communities that are engaged in detecting and resolving difficulties facing them (Kolanchu 2011:24). This is also supported by Babb (2005:11) who argues that the philosophy also cultivates a sense of being in the right place, appreciating variety in others and analogous life prospects. The advantage of using this theoretical framework is that it enables people to speak for themselves on issues of joy and their concerns without fear of intimidation.

### The Biblical concept of disaster preparedness

The basis for mainstreaming disaster danger lessening in the course of theological and religious institutions is motivated by a hermeneutical analysis of the Old Testament text in Genesis 6:14–19 that says:

Make yourself an Ark of cypress wood; make rooms in the ark, and cover it inside and out with pitch … And of every living thing, of all flesh, you shall bring two of every kind into the ark, to keep them alive with you; they shall be male and female.

It is from the text that one discovers that, Noah was warned by God about an impending disaster, and he became one of the earliest religious leaders to be trained in disaster preparedness by God. This, therefore, suggests that as faith leaders and as institutions that are founded on faith, we must always be prepared for disasters because disasters do not wait for us to learn after they occur. We must be ready when disaster strikes. Active participation is called for by God for humanity to take an active role and participate in order to mitigate sufferings during the time of impending disasters.

### Religious institution (United Theological College)s’ potential for disaster management

Gondongwe (pers. comm., 2022) stated that faith-based organisations do not concern themselves in quantifiable handouts. They instead have a mandate to wholistically offer a full package of help to meet physical, emotional and spiritual needs of humanity in critical moments. The UTC was largely involved during the cyclone Idai and COVID-19 pandemic, through provision of shelter for the victims, and quarantine facilities for the patients. Furthermore, faith-based DRM is the practice of discharging tragedy risk administration interventions using spiritual, human, social, economic, financial, physical capital that reside in faith-based communities and institutions to transform societies to a great recovery and resilience against disasters through coordination, collaboration and cooperation with other faith-based institutions and/or other stakeholders. Faith-based institutions are viewed as a resource on their own.

### The role of religious institutions in raising public awareness

In an interview conducted at the UTC, it was deduced that:

Community consciousness is sincere education in that people are conscientized, equipped and alerted to react positively towards taking safe precautions which without education they may not think of. You need to be aware that villagers possess some information and skill in caring and defending themselves against any form of danger. (Gondongwe, pers. comm., 2022)

This is the reason Wisner et al. (2006:331) emphasised that any communiqué to alert danger ought to be in dialogical form to raise the consciousness of the community. Dube and Munsaka (2018:5) aver that in various rising nations, Zimbabwe included there is little information available and thus scant awareness of the community on matters of economic, structural, emotional, healthiness and ecological damage triggered by disasters. As a result, there is an urgent need for religious institutions to embrace and mainstream DRR in their curriculum as a tool to equip church members. They further explain that creating consciousness of disaster threats equip the community to be extra cautious and be proactive rather than being reactive.

The intricacy of tragedy management entails more composite diversified communal systems inclusive of religious leaders. Societies need active participation in making choices that have an impact to their livings. Furthermore, communities led by religious leaders should lead programmes that aim to implement preventive strategies. Equipping the community through education must be the starting point for the ministers of religion who would have graduated from religious institutions such as the UTC. Trained religious leaders become agents that evoke community attention and participation (Kolanchu 2011:48). Community mobilisation is the answer to prevent human tragedies, demolition of buildings and inculcating a spirit of prevention only if awareness is brought to them through the structures of religious organisations that commands a wider coverage compared to other systems like non-governmental organizations (NGO) and government structures themselves.

The constant contact between religious leaders with the residents creates a relaxed atmosphere that enables communities to inquire on issues they do not understand and easily speak their mind compared to political structures, which sometime create an intimidatory atmosphere. Consciousness rising resources should be designed to be comprehended by the local people. This will be attained once the community is engaged throughout the development process. Community consciousness is only attained through involvement of the local churches, civic meetings and institutes. Churches often are left out and least considered as the crucial point for creating consciousness about disaster risk lessening. This was the case in Zimbabwe when COVID-19 started, church leaders were not considered to be among frontline workers such as health workers, uniformed forces and those who were in critical services. Ministers of religion are essential associates in speaking about barricades to the acceptance of well-being and other indispensable amenities, as well as vaccines. According to Maiden (2021):

UNICEF’s Global Faith for Positive Change Initiative recognises the central role and influence of religious leaders in behaviour and social change communication. (p. 3)

Furthermore, public awareness through religious institutions like the UTC can be a useful instrument, particularly when it encompasses valuable material related to the public life, inclusive of exit routes and assemblage points (Mhlanga et al. 2019:6).

Gwenambira, one of the participants during the focus group session, has this to say:

Religious institutions like UTC remain the pinnacle of information dissemination especially on issues to do with disasters. Because of UTC’s ecumenical nature where different denominations are trained and equipped to go into societies around the country, it makes life easier for the nation to benefit from these trained ministers. (Gwenambira, pers. comm., 2023)

From his assessment, it shows that the UTC is strategically positioned to play a pivotal role among other religious institutions to raise public awareness of the entire nation about DRM.

### Knowledge development in religious institutions (United Theological College)

Real-Dato (2009:5) identified the implementation of measures and strategies for religious formal education is the contributing reason that improves the efficiency of emergency administration.. Learning systems at the UTC are aimed at intensifying the ability, receptiveness and expertise of emergency performers and administrations and to unceasingly recover the backup organisation structure completely (Zhou et al. 2011:9). Furthermore, with respect to educational studies planned to equip religious leaders for a precise action and their working tasks, a participant in an interview stated that:

The importance of training and educational courses aimed toward developing general understanding and theoretical knowledge of emergency management. (Gwenambira, pers. comm., 2022)

Additionally:

To heighten their effectiveness, institutional learning arrangements should be implemented both within organizations and at integrated subsystem levels. (Alexander 2003:7)

This is also supported by the Hyogo Framework for Action (2005–2015) that evidently articulates that:

Data, invention and learning must be designed to inculcate a philosophy of protection and pliability at all echelons. Tragedies can be considerably abridged if individuals are properly informed and motivated toward a culture of disaster prevention and resilience, which in turn requires the collection, compilation and dissemination of relevant knowledge and information on hazards, vulnerabilities, and capacities. (Institute for Ocean Management 2007:58)

In addition:

Enabler 2 of the National Disaster Management Framework of 2005 addresses the requirement for the development and implementation of national education and training to capacitate role players through informed scientific research. It also outlines the inclusion of disaster risk management in school curricula. An example that religious leaders are the best educators concerns a clergy with knowledge about floods and tsunamis, who urged evacuation and saved many lives in Muzarabani area in Zimbabwe. (Mudavanhu 2014:8)

Learning institutions are consequently a vital stage for creating consciousness around tragedy risk reduction, particularly religious institutions like the UTC.

## Research results presentation and findings from the study

This discussion is based on the analysed results from the interviews conducted at the UTC and from the students deployed in various corners of Zimbabwe representing six participating churches at the UTC. An interpretation of the results is given, and recommendations are proffered at the end of the article.

### Gender

Any study in the academic circles has to take note of gender representation for a balanced conclusion. An aspect on gender was involved in this study during focus group interviews to find out how many males compared to females are actively participating and likewise to comprehend the wide-ranging viewpoints concerning community consciousness and disaster administration from the perspective of gender representation.

Table 1 shows that 46% of women and 54% of men were active in the focus group research survey. Also men are more active in public awareness activities than women. Even though, statistics favour men compared to women in terms of active participation and public awareness, it is, however, these disparities that should be corrected because women are always available in communities than men who might be away because of other commitments. It was also observed during the study that women are easy to mobilise than men who sometimes ask for reasons why people must assemble. It could be that women are politically influenced by men and are denied chances to participate in gatherings that may not directly benefit them and more so called by other men. In churches, women are always present in more numbers than men at any given gathering. This is attributed by the fact that women sought for divine intervention in challenges they face more than men who sometimes suffer in silence, hence the reason for a small variance in gender depiction. This is supported by Wisner et al. (2006) when he said:

Women are most likely to be at home taking care of young children and this affects their relative vulnerability. Furthermore, men and women’s time and place patterns of daily and seasonal activities differ which may produce inequalities in their exposure to disaster. (p. 239; see also Kolanchu 2011:57)

**TABLE 1:** Participant representation according to gender (*N* = 50).

Participants	*n*	Proportion (%)
Male	27	54
Female	23	46

Consciousness programmes need to be designed in order to encompass these disparities amid male and female. Therefore, the research encourages the inclusion of women as core partners in issues of disaster preparation and execution.

If women are more in churches that are led by religious leaders who might have obtained knowledge and skills of disaster preparedness from religious institutions, then the lives of the women who are at high risk of vulnerability compared men could be saved. If awareness programmes are targeted at religious institutions with religious leaders later disseminating information to their churches, this may help alleviate disaster risk preparedness in communities.

### Age

The aspect of age is critical when it comes to raising awareness to the community. This is attested by Wisner (2006:68) who confirm that in certain circumstances the young ones and the aged are mostly susceptible to the effects of disasters. This is a true to what happened in Chimanimani during cyclone Idai between 04 March 2019 to 21 March 2019. The young and the aged are more susceptible to the effects of disasters because of their bodily powerlessness to react quickly for safety. Conscientisation programmes must therefore embrace the age aspect including age component in the focus group study questions. Age categorisation helps in creating a thoughtful consideration of community responsiveness in diverse age clusters. The study purposively selected participants who were 18 years and above because it is alleged that communal responsiveness can disrupt barricades of age by transporting data from the young generation to the old generation and vice versa.

The results presented in Table 2 illustrate that the uppermost proportion of participants (38.4%) comprised of young people between the age of 18–25 years followed by young adults between 26 and 35 years (28.0%) and least representation (6%) by adults above 56 years. The study outcomes replicate correct picture of people in the diverse clusters in the UTC community giving their autonomous opinions, even those over 56 years. It can also be deduced that the age group between 18 to 45 years are still active, and they are actively participating in churches where religious leaders preach every Sunday, or any other days scheduled for worship. Most of the religious leaders who are recruited at UTC are below the age of 35 years as reflected by one denomination that has a recruitment age limit of 35 years maximum at candidature (MCZ Minutes of Conference 2021:11). When disaster risk preparedness is taught at UTC, it will help to target people of all ages who are members of the churches where these trained religious leaders go to minister across the country. This research shows that when UTC involved its trained pastors to spearhead awareness programmes during its engagement in community programmes most participants who voluntarily came for awareness programmes were those in the active age category. One of the participant argued that creating awareness programmes for those who are in their active age will help the nation to curb disasters because it is these young generations that do not know how to react during the times of disasters. Thus to save lives, information dissemination must target both the youths and the elderly.

**TABLE 2:** Participant representation according to age (*N* = 50).

Stage of development (in years)	*n*	Proportion (%)
18–25	19	38.4
26–35	14	28.0
36–45	9	18.0
46–55	5	9.6
> 56	3	6.0

### Knowledge of risk involving disaster actions

Information is understood as ‘the detail or state of comprehending rather with a significant notch of awareness via practice, memory or interaction’ (Kolanchu 2011:60). During a test case community awareness workshop initiated by the UTC for its former students who graduated from the UTC, this inquiry is necessary in order to assess if the contestants in the research possess information regarding the need for community consciousness. Furthermore, it also differentiates the drive of this research that emphasises additionally on community consciousness in relation to DRM instead of focussing on contamination that attracts attention of most communities inclusive of the local authorities.

The responses to the question about information dissemination in risk-involving actions (cf. Table 3), in Table 3 shows that 34% of the participants who are former students of the UTC had meagre information of risk involving disaster actions, whereas 50% possess reasonable information and at least 16% possess adequate information. These answers propose that a sizable quantity of religious leaders do possess inadequate to reasonable knowledge or understanding of disasters. The replies also submit that religious leaders who pass through the UTC have inadequate information and skills on issues of tragedy risk administration, and this creates a need for more education. The scenario indicates that a number of participants passed through the UTC before the DRM was an issue of prevalence. Therefore, there is a need to conduct refresher courses in line with DRM in order to create awareness for the former students.

**TABLE 3:** Participant representation according to information dissemination of risk-involving actions (*N* = 50).

Variable	*n*	Proportion (%)
Poor	17	34
Moderate	25	50
Good	8	16

### Consciousness of perils

The question on consciousness of dangers is critical to assess how the religious leaders who are former students of the UTC came to gain knowledge about the hazards predominant in their community. Furthermore, the question seeks to determine the awareness of the contestants about the issues that disturb them and their active community involvement to mitigate these issues.

As shown in Table 4, 34% of the participants got information about the dominant hazards via public assemblies, 32% through mass media, 12% via formal training, and 22% through supplementary sources, for instance, church gatherings, conversations and some at their work places. The outcomes show that participants are worried around their security and welfare, and they are actively engaged in society activities. This shows that information gathering throughout risk valuation must include community contribution comprising indigenous knowledge and historical accounts. The data also show that little activity is done through church activities; however, this is the platform where many people are found cutting across gender and age. More attention therefore, should be paid to equipping faith leaders through mainstreaming of disaster risk preparedness courses at the UTC.

**TABLE 4:** Participant representation according to consciousness of perils (*N* = 50).

Question Variable				*n*	Proportion (%)
Training	6	12
Media awareness	16	32
Community meetings	17	34
Other (Church, Political gatherings)	11	22

Note: Question asked: How did you get to know about the hazards?

### Church and community involvement

The disaster peril lessening strategy and administration in Zimbabwe needs emphasis on the participation of the community in the management of disasters (Mavhura 2017:6). Organization for Economic Co-operation and Development (2003:79) states that:

To the extent that the public is informed about the risks in their community, they are more likely to participate in decision-making processes and take steps to help reduce the risks. This part of the questionnaire sought to establish what the participants have done to address disaster management issues in their area. It also probed as to which programmes the participants were involved in to address the hazards.

The results in Table 5 show that from the religious leaders who are former students of the UTC 30% participated in raising consciousness; 22% participated in education and training and 18% participated in voluntary work such as being coopted in disaster risk committees at local communities. Nevertheless, 30% of the participants indicated that they did not take any action about the information. Be that as it may, the results show that 70% of religious leaders took part in one way or the other in community engagements to conscientise the people. Furthermore, involvement of the less privileged groups such as youths and women should be given a priority to expand the quality and raise the livelihood of community empowerment and engagement in tragedy reduction activities especially when initiated through church activities, which are non-partisan and accommodate all the sundry. These indicators drawn from the research show that UTC should play a big part in mitigation reduction of disasters and their risks in Africa and particularly in Zimbabwe. Having looked at the responses, it is now fitting to present the opportunities and challenges bedevilling the UTC in trying to mainstream disaster risk management in the curricula.

**TABLE 5:** Participant representation according to church and public participation (*N* = 50).

Question variable	*n*	Proportion (%)
Awareness	15	30
Education and training	11	22
Volunteering work	9	18
Other	15	30

Note: Question asked: How did one get to participate in these issues?

## Conclusion and recommendations

From the discussion above, one can deduce that religious convictions contribute a critical role in communal development, particularly in Africa. This gives an added advantage for religion to contribute a key part in disaster danger administration, especially when religious leaders are equipped and well prepared for DRM. In most disasters happening in the community, religious leaders are one of the first groups to respond in mitigation and continue to be visible or available post-disaster. The focus group also deduced that most communities, especially in Africa, provide divine or spiritual clarification to nearly every disaster. As alluded elsewhere in this study, one may not deny the fact that spiritual leaders are crowd pullers as compared to politicians, and they are visible in every community.

Furthermore, it has been learnt that religious leaders offer their adherents spiritual and moral guidance during the time of crisis; hence, it is to the advantage of religious institutions to be involved in disaster management without raising any suspicion from the community. Accordingly, it has been concluded during the study that religious leaders offer help and support in times of distress or discomfort, they offer counselling and material needs of the people. This is spiritual and emotional care. Religious leaders are also held in high esteem by the adherents and sometimes their words can be held and listened to more carefully than scientific information:

In Zimbabwe prophets such as Makandiwa has divided Zimbabweans after contradicting himself on COVID-19 by backtracking on COVID vaccines as he urges loyalists not to get vaccinated. (Ndoro 2021:4)

During the COVID-19 epidemic, roughly intrigue philosophers in Christianity have construed masks and vaccines as the mark of the beast. Throughout COVID-19, most spiritual leaders were not regarded as part of the front-line workers by most countries up to a time when things did not go well. In Zimbabwe, particularly, people refused to take vaccines, and it was at this time that the Zimbabwean government realised the need for spiritual leaders to be at the fore front. This is because religion contributes a vital part in societal growth, specifically in Africa. Religious leaders perform many rituals varying from faith-to-faith, for example, healing sessions.

Additionally, religious leaders spearhead disaster relief response even in hard-to-reach areas. They are frontline workers who taught and provided ministry of presence in the country that was grossly affected by COVID-19 and experienced other disasters like cyclone Idai. Religious leaders pray and help adherents to pray and encourage commitment to the faith. Through religious institutions they are affiliated to, they have the capacity to mobilise resources. In a nutshell, the UTC being an ecumenical institution can mainstream issues of tragedy risk administration in its curricula towards capacitating religious leaders beforehand and after the disaster because it has a religiously relevant and diversity advantage as well as a God given mandate derived from scripture (John 10 vs 10). Finally, the Church is usually relevant on response and recovery phases, and it is closer to the people.

However, there are also some challenges observed during the study that may hinder religious institutions to mainstream DRM in their curricula, one of which is lack of resources. This may adversely affect the UTC specifically; unless it is well resourced it may face hurdles along the way. Another challenge is that they may be seen to be competing with government systems in the issues of disaster risk management and hence may not be effective enough in the community. Religious institutions and their leaders are always sidelined by government, which at first saw religious leaders as not part of frontline workers but only to reverse later. The major obstacle is lack of human resources to spearhead the training.

Be that as it may, one may conclude by advocating for a buy in of theological and religious institutions to mitigate and mainstream DRM from an informed point of view. This can be done through the development of disaster management module, which can be a stand-alone course or that can be mainstreamed in already existing programmes. The trained ministers can integrate the acquired knowledge through engagement with parishioners in class meetings and/or home meetings, preaching counselling series, Bible study, funerals, weddings and in liturgy. Religious institutions are challenged to accept the challenge and start something towards disaster risk management, develop modules and integrate disaster risk management within current theological studies for prevention, mitigation and effective response to current and future disasters within communities.

Conclusively, lessons that can be drawn from the study as motivation for religious institutions to be prepared for disasters are that:

- Disasters happen all the time in human societies and in the natural environment.
- Leaders in general must always be prepared to minimise the effect of tragedies on their societies and other communities also.
- Faith leaders are community leaders; hence they must ready themselves to drive disaster responses in their communities.
- Saving lives, of human beings and all other living creatures, must be the primary goal of any DRM effort.
- Disaster preparedness must be built before disasters strike our communities; during the disaster, there is no time to learn, but only time to execute our plans.
- Listen attentively to warnings and react accordingly, warn others, prepare yourself and prepare others. It is our duty to inform and warn others and to make the necessary plans, most disasters catch us unprepared because we refused to prepare when we were warned.
- As faith leaders and actors from a faith background, we have an obligation to be leaders before, during and after disasters have visited our communities.

## Footnotes

1 The UTC was founded in 1954 as an institution of the Methodist Church in Zimbabwe situated on part of the Epworth Mission Farm and named Epworth College, it became an ecumenical Protestant Seminary when in 1955 the Methodist Church in Zimbabwe, the United Methodist Church, the United Church of Christ, the Evangelical Lutheran Church in Zimbabwe, the Uniting Presbyterian Church of Southern Africa, African Methodist Episcopal Church, Independent African Church and the United Congregational Church of Southern Africa (hereinafter called the ‘participating churches’) resolved to co-operate in sponsoring the College for the training of pastors and other Church workers. In 1976, the name was changed to UTC. The UTC is an ecumenical institution whose purpose is to train quality Pastors and teachers of the Christian Gospel for Churches in Zimbabwe and the region (United Theological College Handbook 2022:3)
